# Supplementary material for: A systematic review of evidence on employment transitions and weight change by gender in ageing populations
Source: PLoS One. 2022 Aug 18;17(8):e0273218. doi: 10.1371/journal.pone.0273218 (PMC9387864; doi:10.1371/journal.pone.0273218)
Supplement: S1 Checklist — (DOCX) [file pone.0273218.s001.docx]

| **Section and Topic** | **Item #** | **Checklist item** | **Location where item is reported** |  |
| --- | --- | --- | --- | --- |
| **TITLE** | | |  |  |
| Title | 1 | Identify the report as a systematic review. | Pg. 1 | *A* ***systematic review*** *of evidence on employment transitions and weight change by gender in ageing populations* |
| **ABSTRACT** | | |  |  |
| Abstract | 2 | See the PRISMA 2020 for Abstracts checklist. | Pg. 2 |  |
| **INTRODUCTION** | | |  |  |
| Rationale | 3 | Describe the rationale for the review in the context of existing knowledge. | Pg. 4-6 | “However, these results have not been consistently supported in the literature [17,18]. It has been noted that change in body weight may depend on gender [12], occupation type [14], and changes in physical activity levels following a transition [14].  One additional post-transition behaviour change that may also be important in the effect of employment transitions on body weight is sleep [19–25].”  and  “In sum, there is a potential mediating role that sleep may play in the impact of employment transitions on subsequent body weight gain. Sleep may contribute to explaining the heterogeneity that appears to exist in the literature on job-loss and retirement and body weight gain in older adults, yet it has not received as much attention as physical activity [14,18], or other modifiable health behaviours [15,16].” |
| Objectives | 4 | Provide an explicit statement of the objective(s) or question(s) the review addresses. | Pg. 6 | “To address this gap, this review has two objectives: (1) to systematically search the existing literature on the impact that employment transitions have on body weight by women and men among middle-aged and older adults, and (2) to examine whether sleep confounds, mediates, or modifies the effect that employment transitions have on body weight in the identified studies.” |
| **METHODS** | | |  |  |
| Eligibility criteria | 5 | Specify the inclusion and exclusion criteria for the review and how studies were grouped for the syntheses. | Pg. 8 | See section: **Criteria**  “A study was eligible for inclusion if it examined the longitudinal association of employment transition (e.g., a change in employment status between two time-points) and subsequent body weight outcomes in adults aged 50 years of age and older. Studies were excluded if the exposure of interest was employment status at only single point in time as the objective of this review is to examine how changes to employment status may be associated with body weight outcomes. Examples of changes in employment status are job-loss (employed at baseline of study and unemployed at follow-up) and retirement (employed at baseline of study and retired and not working at follow-up). No restrictions were placed on how studies defined the transitions or body weight outcomes. Additional exclusion criteria include: cross-sectional design; qualitative studies; broad age groups with no stratification of results (e.g., 18 years to 65 years); non-body weight outcomes; and, clinical populations. Studies that also included an analysis with sleep parameters were considered for our second objective; however, the absence of sleep as a variable in the analysis was not a criterion for exclusion.” |
| Information sources | 6 | Specify all databases, registers, websites, organisations, reference lists and other sources searched or consulted to identify studies. Specify the date when each source was last searched or consulted. | Pg. 7-8 | See section: **Search and data sources** and **Screening**  “Journal articles were systematically searched using eight bibliometric databases (Ovid MEDLINE/PubMed, Scopus, PsycINFO, Web of Science, Embase, EconLit, CINAHL, and Applied Social Sciences Index and Abstracts).”  and  “Searches were performed by ACTT, VAS, SJ, and TYL between June and July 2021.”  and  “Records were removed based on exclusion criteria and eligible full-texts were retrieved and screened for inclusion and reference tracing” |
| Search strategy | 7 | Present the full search strategies for all databases, registers and websites, including any filters and limits used. | Pg. 7 and Table S1 | See section: **Search and data sources**  “Table 1 contains the search terms used in the databases after consulting with a reference librarian. Free-text thesaurus and MeSH terms were used with Boolean operators to capture the concepts of “middle and older age”, “employment”, “transition”, and “body weight”. No restrictions were placed on publication date, country, or original publication language. Screening, assessment, and inclusion of studies published in English, French, and Chinese.”  Search strategies for all databases are in supplementary Table S1. |
| Selection process | 8 | Specify the methods used to decide whether a study met the inclusion criteria of the review, including how many reviewers screened each record and each report retrieved, whether they worked independently, and if applicable, details of automation tools used in the process. | Pg. 8 | See section: **Screening**  “Two reviewers (ACTT and VAS, SJ, or TYL) screened titles and abstracts independently. Records were removed based on exclusion criteria and eligible full-texts were retrieved and screened for inclusion and reference-tracing adhering to the same division of work between two independent reviewers. All disagreements were managed with discussion among reviewers and resolved by consensus.” |
| Data collection process | 9 | Specify the methods used to collect data from reports, including how many reviewers collected data from each report, whether they worked independently, any processes for obtaining or confirming data from study investigators, and if applicable, details of automation tools used in the process. | Pg. 9-10 | See section: **Risk of bias assessment, data extraction, and analysis**  “VAS, SJ, and TYL extracted data from studies into a standardised evidence table: author; objectives; study time frame; setting; research design; sample characteristics; description of exposure; description of outcome; time to follow-up; job-types specified; and, body weight results. Where relevant, gender and job-type stratified results are also populated into the results field. ACTT reviewed the extracted data and provided feedback to the three extractors during the process. A narrative synthesis was used to summarize the findings.” |
| Data items | 10a | List and define all outcomes for which data were sought. Specify whether all results that were compatible with each outcome domain in each study were sought (e.g. for all measures, time points, analyses), and if not, the methods used to decide which results to collect. | Pg. 9-10 | Same as above. |
|  | 10b | List and define all other variables for which data were sought (e.g. participant and intervention characteristics, funding sources). Describe any assumptions made about any missing or unclear information. | Pg. 9-10 | Same as above. |
| Study risk of bias assessment | 11 | Specify the methods used to assess risk of bias in the included studies, including details of the tool(s) used, how many reviewers assessed each study and whether they worked independently, and if applicable, details of automation tools used in the process. | Pg. 9-10 | See section: **Risk of bias assessment, data extraction, and analysis**  “Studies were appraised using the Effective Public Health Practice Project (EPHPP) tool given its suitability for assessing quantitative studies based on observational data [34]. The EPHPP quality assessment tool contains 21-items with sections that cover components such as selection bias, study design, confounding, data collection, and analysis considerations. Of the 21 items, 11 are directly relevant in assigning a global rating of “strong”, “moderate”, or “weak” to our included studies. ACTT and VAS independently assessed study quality and initial assessments were compared, with final ratings established by consensus.” |
| Effect measures | 12 | Specify for each outcome the effect measure(s) (e.g. risk ratio, mean difference) used in the synthesis or presentation of results. | Pg. 11 and Table S3 | “Eight of the studies used change in body weight index (BMI) between time points as their body weight outcome [14,16,50–55]. Additional outcomes included odds of weight change [17,56], odds or risk of becoming overweight or obese [52,55], and annualized weight change [12].” |
| Synthesis methods | 13a | Describe the processes used to decide which studies were eligible for each synthesis (e.g. tabulating the study intervention characteristics and comparing against the planned groups for each synthesis (item #5)). | N/A | All included studies that met inclusion criteria were eligible for synthesis. |
|  | 13b | Describe any methods required to prepare the data for presentation or synthesis, such as handling of missing summary statistics, or data conversions. | Pg. 10 | See section: **Risk of bias assessment, data extraction, and analysis**  “VAS, SJ, and TYL extracted data from studies into a standardised evidence table: author; objectives; study time frame; setting; research design; sample characteristics; description of exposure; description of outcome; time to follow-up; job-types specified; and, body weight results. Where relevant, gender and job-type stratified results are also populated into the results field. ACTT reviewed the extracted data and provided feedback to the three extractors during the process.” |
|  | 13c | Describe any methods used to tabulate or visually display results of individual studies and syntheses. | Pg. 10, 20 | Same as above  and  “Table 5 provides a summarised overview of data extracted from included studies with respect to the direction of change in the body weight outcome by employment transition, gender, occupation type, and comparator (pre-post or against a control). Detailed extraction of included study results and definitions of occupation types are provided as online supporting information (Tables S1 and S2).” |
|  | 13d | Describe any methods used to synthesize results and provide a rationale for the choice(s). If meta-analysis was performed, describe the model(s), method(s) to identify the presence and extent of statistical heterogeneity, and software package(s) used. | Pg. 8,10 | “No restrictions were placed on how studies defined the transitions or body weight outcomes.”  and  “A narrative synthesis was used to summarize the findings.” |
|  | 13e | Describe any methods used to explore possible causes of heterogeneity among study results (e.g. subgroup analysis, meta-regression). | Pg. 27-31 | See section: **Body weight outcomes of employment transitions by occupation type and by gender**  and  See section: **Role of sleep and other health behaviours as potential mediators** |
|  | 13f | Describe any sensitivity analyses conducted to assess robustness of the synthesized results. | N/A – narrative synthesis | No sensitivity analyses were conducted for our narrative synthesis. |
| Reporting bias assessment | 14 | Describe any methods used to assess risk of bias due to missing results in a synthesis (arising from reporting biases). | N/A | Acknowledged publication bias in limitations. |
| Certainty assessment | 15 | Describe any methods used to assess certainty (or confidence) in the body of evidence for an outcome. | N/A – narrative synthesis | No certainty assessment was conducted for our narrative synthesis. We discuss the mixed results in the Discussion.  “Findings from pre-post analyses suggest women tend to experience weight gain [12,53], and men may experience either weight loss [16,53], or weight gain following retirement [12,16,18], but there is much uncertainty associated with these findings due to the few pre-post only studies available and even fewer studies that included women in the study sample.”  “However, weight gain was only found among studies using self-reported outcomes [17,51], and not among studies using objectively measured anthropometry [12,54]. The literature also appears inconsistent in reported effects of retirement for among men [12,17,51,54,56]. The evidence on gender differences, however, is not clear given that both the increases and decreases in the weight of women and men were not consistently found across different studies, potentially owing to differences in how studies defined their outcomes [12,14,17,51,54,56]. Moreover, we also found that occupation type modified the effect of retirement on weight, but results were more clear for the weight gain after retirement from physically demanding occupations [14,17,52,56], than the reported weight change following retirement from sedentary jobs [14,17,52,56]. Finally, job-loss appears to lead to more weight gain than continuous employment [12,16]; however, more studies are required to ascertain gender differences.” |
| **RESULTS** | | |  |  |
| Study selection | 16a | Describe the results of the search and selection process, from the number of records identified in the search to the number of studies included in the review, ideally using a flow diagram. | Pg. 10-11 | “The results of the database searches are presented in a flow diagram in Figure 1. Database searches and citation tracing identified 6,001 unique records after duplicates were removed (n= 1,754). In total, 27 original research articles remained after title and abstract screening and were read in full. Of the 27, 12 were eligible for inclusion and were assessed for risk of bias, extracted from, and synthesized.” |
|  | 16b | Cite studies that might appear to meet the inclusion criteria, but which were excluded, and explain why they were excluded. | Pg. 11 | “Fifteen studies were excluded after full-text review. Ten studies were excluded because results were not relevant to the outcomes of interest (body weight) [36–45]. Three studies used employment status at a single time point and thus, did not measure the exposure of interest (employment transition) [46–48]. The two remaining studies investigated body weight as a determinant of retirement decisions and did not have results relevant to the desired direction of association [49]; or was a protocol document [50].” |
| Study characteristics | 17 | Cite each included study and present its characteristics. | Table 3 |  |
| Risk of bias in studies | 18 | Present assessments of risk of bias for each included study. | Table 4 |  |
| Results of individual studies | 19 | For all outcomes, present, for each study: (a) summary statistics for each group (where appropriate) and (b) an effect estimate and its precision (e.g. confidence/credible interval), ideally using structured tables or plots. | Table S3 |  |
| Results of syntheses | 20a | For each synthesis, briefly summarise the characteristics and risk of bias among contributing studies. | Pg. 11-12, 16 | See section: **Characteristics of included studies**  “The characteristics of the included studies are presented in Table 3. All 12 included studies analyzed the […]”  and  See section: **Quality of included studies**  **Quality of included studies**  “Two studies were rated as weak [53,54]; a majority of the studies (n= 8) were considered moderate in quality [12,14,16–18,51,55,57]; and two were rated as strong [52,56] (table 4). The reasons for the weak rating included […]” |
|  | 20b | Present results of all statistical syntheses conducted. If meta-analysis was done, present for each the summary estimate and its precision (e.g. confidence/credible interval) and measures of statistical heterogeneity. If comparing groups, describe the direction of the effect. | N/A – narrative synthesis | We synthesized evidence narratively. |
|  | 20c | Present results of all investigations of possible causes of heterogeneity among study results. | Pg. 27-31 | See section: **Body weight outcomes of employment transitions by occupation type and by gender**  and  See section: **Role of sleep and other health behaviours as potential mediators** |
|  | 20d | Present results of all sensitivity analyses conducted to assess the robustness of the synthesized results. | N/A – narrative synthesis | No sensitivity analyses were conducted for our narrative synthesis. |
| Reporting biases | 21 | Present assessments of risk of bias due to missing results (arising from reporting biases) for each synthesis assessed. | N/A | Acknowledged publication bias in limitations. |
| Certainty of evidence | 22 | Present assessments of certainty (or confidence) in the body of evidence for each outcome assessed. | N/A – narrative synthesis | No certainty assessment was conducted for our narrative synthesis. See item 15. |
| **DISCUSSION** | | |  |  |
| Discussion | 23a | Provide a general interpretation of the results in the context of other evidence. | Pg. 32-33 | “The results of the included studies generally fall into two categories: pre-post changes in weight within employment transitions or changes in weight for employment transitions relative to a comparison group (i.e., those who remained employed or did not retire). Findings from pre-post analyses suggest […]” |
|  | 23b | Discuss any limitations of the evidence included in the review. | Pg. 36-37 | “Thirdly, publication bias may be present. As this review has found, null results have been observed when considerations around sex/gender, occupation type, and direction of body weight change are not adequately addressed. Lastly, the studies identified were mostly based in high-income countries. With respect to differences in labour force markets, cultural norms of working and ageing, health and pension systems, we expected the impacts of employment transitions on body weight to vary by countries. This highlights another important gap in the literature that future research should address.” |
|  | 23c | Discuss any limitations of the review processes used. | Pg. 36 | “The first limitation is the difficulty in identifying studies that investigated “employment transitions” as this did not appear to be a term that was used explicitly in many articles. Often, a thorough reading of the methods section was required to verify whether a study truly examined a change in employment status over, at least, two points of time. However, the use of a dual reviewer approach and practice screenings would have mitigated most of this problem. However, some studies may still have been missed. A second limitation is related to our quality appraisal tool. Observational studies that used either pre-post or pre-post with comparison were both given the same rating under the design domain, despite the latter offering potentially more robust findings when well-executed. Assigning a different score to each of the studies would have helped to further differentiate the quality of the evidence.” |
|  | 23d | Discuss implications of the results for practice, policy, and future research. | Pg. 37 | “The findings from our systematic review suggest that the effect of employment transitions on body weight outcomes in middle-aged and older adults differ by gender, occupation types and countries. The existing body of literature brings to attention the large research gaps. Potential key areas of focus in future studies include: the causal relationship and sleep as a third variable, the effect of job-loss as opposed to retirement, whether the effect of employment transitions differ by levels of socioeconomic status or across comorbidities related to overweight or obesity and, the impact of alternative operational definitions of weight change on study results. More rigorous evidence synthesis methods are also needed in future literature reviews to address study and topic complexity.” |
| **OTHER INFORMATION** | | |  |  |
| Registration and protocol | 24a | Provide registration information for the review, including register name and registration number, or state that the review was not registered. | N/A – did not register | Protocol was not registered. |
|  | 24b | Indicate where the review protocol can be accessed, or state that a protocol was not prepared. | N/A – did not register | Protocol was not registered. |
|  | 24c | Describe and explain any amendments to information provided at registration or in the protocol. | N/A – did not register | Protocol was not registered. |
| Support | 25 | Describe sources of financial or non-financial support for the review, and the role of the funders or sponsors in the review. | To be entered to submission system |  |
| Competing interests | 26 | Declare any competing interests of review authors. | To be entered to submission system |  |
| Availability of data, code and other materials | 27 | Report which of the following are publicly available and where they can be found: template data collection forms; data extracted from included studies; data used for all analyses; analytic code; any other materials used in the review. | Online Supporting Information |  |

*From:*  Page MJ, McKenzie JE, Bossuyt PM, Boutron I, Hoffmann TC, Mulrow CD, et al. The PRISMA 2020 statement: an updated guideline for reporting systematic reviews. BMJ 2021;372:n71. doi: 10.1136/bmj.n71

For more information, visit: <http://www.prisma-statement.org/>
