# Supplementary material for: A systematic review of evidence on employment transitions and weight change by gender in ageing populations
Source: PLoS One. 2022 Aug 18;17(8):e0273218. doi: 10.1371/journal.pone.0273218 (PMC9387864; doi:10.1371/journal.pone.0273218)
Supplement: S1 Table — (DOCX) [file pone.0273218.s002.docx]

**S1 Table. Search strings used in each bibliographic database.**

| Search # | Search string |
| --- | --- |
| **ASSIA** | |
| #1 | (weight OR bmi OR body mass index OR adipos* OR “met* syndrome” OR “cardiovascular disease”) AND (change OR loss OR transition OR terminat* OR dismiss* OR lay-off OR reduc* OR become* OR enter* OR adjust*) AND (employ* OR job* OR unemploy* OR work* OR retir*) AND (Old* adult* OR ag*ing OR aged OR elder* OR geriatric* OR senior) |
| **CINAHL Complete** | |
| #1 | MW aging OR MW aged |
| #2 | AB (old* adult* OR ag#ing OR aged OR elder* OR geriatric* OR senior) |
| #3 | S1 OR S2 |
| #4 | AB (employ* OR job* OR unemploy* OR work* OR retir*) |
| #5 | AB (change OR loss OR transition OR terminat* OR dismiss* OR lay-off OR reduc* OR becom* OR enter* OR adjust*) |
| #6 | AB (weight OR bmi OR body mass index OR adipos* OR “met* syndrome” OR “cardiovascular disease”) |
| #7 | MW Adiposity OR MW Body Weight OR MW Metabolic Syndrome OR MW cardiovascular disease |
| #8 | S6 OR S7 |
| #9 | S3 AND S4 AND S5 AND S8 |
| **EconLit** | |
| #1 | (weight OR bmi OR body mass index OR adipos* OR “met* syndrome” OR “cardiovascular disease”) AND (change OR loss OR transition OR terminat* OR dismiss* OR lay-off OR reduc* OR become* OR enter* OR adjust*) AND (employ* OR job* OR unemploy* OR work* OR retir*) AND (Old* adult* OR ag*ing OR aged OR elder* OR geriatric* OR senior) |
| **Embase and Ovid MEDLINE** | |
| #1 | (old* adult* or ag?ing or aged or elder* or geriatric* or senior).tw. |
| #2 | (weight or bmi or body mass index or adipos* or "met* syndrome" or "cardiovascular disease").tw. |
| #3 | ((change or loss or transition or terminat* or dismiss* or lay-off or reduc* or becom* or enter* or adjust*) adj5 (employ* or job* or unemploy* or work* or retir*)).tw. |
| #4 | Aging/ |
| #5 | Aged/ |
| #6 | Adiposity/ |
| #7 | Body Weight/ |
| #8 | Metabolic Syndrome/ |
| #9 | Cardiovascular Diseases/ |
| #10 | 1 or 4 or 5 |
| #11 | 2 or 6 or 7 or 8 or 9 |
| #12 | 3 and 10 and 11 |
| **PsycInfo** | |
| #1 | AB (old* adult* OR ag?ing OR aged OR elder* OR geriatric* OR senior) |
| #2 | AB (employ* OR job* OR unemploy* OR work* OR retir*) |
| #3 | AB (change OR loss OR transition OR terminat* OR dismiss* OR lay-off OR reduc* OR becom* OR enter* OR adjust*) |
| #4 | AB (weight OR bmi OR body mass index OR adipos* OR “met* syndrome” OR “cardiovascular disease”) |
| #5 | (S1 AND S2 AND S3 AND S4) |
| **Scopus** | |
| #1 | (old* AND adult* OR ag*ing OR aged OR elder* OR geriatric* OR senior) |
| #2 | (employ* OR job* OR unemploy* OR work* OR retir*) |
| #3 | (change OR loss OR transition OR terminat* OR dismiss* OR lay-off OR reduc* OR becom* OR enter* OR adjust*) |
| #4 | (weight OR bmi OR body AND mass AND index OR adipos* OR “met* syndrome” OR “cardiovascular disease”) |
| #5 | 1 AND 2 AND 3 AND 4 |
| **Web of Science** | |
| #1 | (old* AND adult* OR ag$ing OR aged OR elder* OR geriatric* OR senior) (topic) |
| #2 | (employ* OR job* OR unemploy* OR work* OR retir*) NEAR/5 (change OR loss OR transition OR terminat* OR dismiss* OR lay-off OR reduc* OR becom* OR enter* OR adjust*) (topic) |
| #3 | (weight OR bmi OR body AND mass AND index OR adipos* OR “met* syndrome” OR “cardiovascular disease”) (topic) |
| #4 | 1 AND 2 AND 3 |
