# Supplementary material for: A systematic review of evidence on employment transitions and weight change by gender in ageing populations
Source: PLoS One. 2022 Aug 18;17(8):e0273218. doi: 10.1371/journal.pone.0273218 (PMC9387864; doi:10.1371/journal.pone.0273218)
Supplement: S2 Table — (DOCX) [file pone.0273218.s003.docx]

**S2 Table. Details of employment categories in included studies.**

| **Author / source** | **Description of job types studied** | **Classification of results into “Active” or “Sedentary” or “All”** |
| --- | --- | --- |
| Morris et al. 1992 [16]  [ProQuest] | **Manual work:** respondents were categorized based on whether their occupation indicates manual work or non-manual work. | **All only:** no stratification of results by job type |
| Nooyens et al. 2005 [18]  [MEDLINE] | **Sedentary job**: respondent described their occupation as sedentary  **Active job:**  respondent described their occupation as requiring standing, manual work, or heavy manual work. | **Active and Sedentary:** results are from corresponding categories |
| Forman-Hoffman et al. 2008 [17]  [MEDLINE] | **White Collar:**  Managerial specialty operation, professional specialty operation/ technical support, sales, clerical/ administrative support, and health services jobs  **Blue Collar:**  Service, mechanics and repair, construction trade and extracting, precision production, and operator jobs  **Other/Farmer/ Military:** farming/forestry/ fishing and armed forces | **Active:** results are from blue collar and other/farmer/military categories  **Sedentary:** results are from white collar category |
| Zheng 2008 [14]  [ProQuest] | **Sedentary occupation:** ≤1 physical demands based on the Dictionary of Occupation Titles (DOT).  **Physically demanding occupation:**  >1 physical demands based on the Dictionary of Occupation Titles (DOT). | **Active:** results are from physically demanding category  **Sedentary:** results are from sedentary category |
| Chung et al. 2009 [52]  [MEDLINE] | Jobs were classified based on definitions from the Dictionary of Occupation Titles and Standard Occupation Classification Systems:  **Sedentary:**  “light” strength occupations  **Physically Demanding:**  “medium”, “heavy”, or “very heavy” strength occupations | **Active:** results are from physically demanding category  **Sedentary:** results are from sedentary category |
| Gueorguieva et al. 2011 [55]  [Hand search] | **Occupations:** respondents were categorized based on reported occupation title into four groups:  (1) **Professional and managerial**  (2) **Sales, clerical, and admin.**  (3) **Service** (e.g. food preparation, health service, personal care service, private household services)  (4) **Other blue-collar** (e.g., construction, machine operators, laborers, forestry) | **Active:** results are from service and other blue-collar categories  **Sedentary:** results are from professional and managerial; and, sales, clerical, and admin categories |
| Monsivais et al. 2015 [12]  [MEDLINE] | **Occupational class:** respondents who held professional or managerial, or technical professions. | **All only:** no stratification of results by job type |
| Godard 2016 [56]  [Hand search] | **Strenuous jobs:** respondents indicated that they agreed or strongly agreed that their job was physically demanding.  **Sedentary jobs:** respondent indicated that they disagreed or strongly disagreed that their job was physically demanding. | **Active:** results are from strenuous jobs category  **Sedentary:** results are from sedentary jobs category |
| Stenholm et al. 2017 [53]  [MEDLINE] | **Sedentary jobs**: sitting at work and no heavy work  **Diverse Jobs**: no sitting at work and no heavy work  **Physically heavy jobs**: no sitting at work but heavy work | **Active:** results are from physically heavy and diverse jobs categories  **Sedentary:** results are from sedentary jobs category |
| Syse et al. 2017 [57]  [PsycINFO] | **Sector of employment:** Public employees versus private employees | **All only:** sector does not indicate active or sedentary job types |
| Feng et al. 2020 [54]  [MEDLINE] | None defined | **All only:** no job types defined |
| Pedron et al. 2020 [51]  [MEDLINE] | None defined | **All only:** no job types defined |
