# Supplementary material for: A systematic review of evidence on employment transitions and weight change by gender in ageing populations
Source: PLoS One. 2022 Aug 18;17(8):e0273218. doi: 10.1371/journal.pone.0273218 (PMC9387864; doi:10.1371/journal.pone.0273218)
Supplement: S3 Table — (DOCX) [file pone.0273218.s004.docx]

**S3 Table. Results of included studies by sex/gender.**

| **Source** | **Results for females/women: Body Weight** | **Results for males/men: Body Weight** |
| --- | --- | --- |
| Morris et al. 1992 [16] | Not applicable. | **Continuously employed:**  2.1% lost >10% in weight  5.0% gained >10% in weight  **Discontinuously employed:**  2.2% lost >10% in weight  5.0% gained >10% in weight  **Unemployed not due to illness:**  2.8% lost >10% in weight  7.1% gained >10% in weight*  **Retired not due to illness:**  1.4% lost >10% in weight  7.5% gained >10% in weight*  *statistically different from continuously employed (p<0.05)  No stratification by manual/non-manual jobs. |
| Nooyens et al. 2005 [18] | Not applicable. | **Sedentary, continue working**:  *Weight*: +0.24 kg/year (0.06, 0.41)  *Waist circumference*: +0.31 cm/year (0.09, 0.53)  **Sedentary, retired**:  +0.08 kg/year (-0.13, 0.30)*  +0.23 cm/year (-0.04, 0.50)^#^  **Active, continue working**:  +0.37 kg/year (0.19, 0.55)  +0.54 cm/year (0.31, 0.77)  **Active, retired**:  +0.42 kg/year (0.17, 0.67)*  +0.77 cm/year (0.46, 1.08)^#^  * statistically different from each other (p<0.10)  ^#^ statistically different from each other (p<0.05) |
| Forman-Hoffman et al. 2008 [17] | **White collar, retirement compared to continued working:**  *Weight loss* ***≥****5%:* OR 1.13 (0.89, 1.43)  *Weight gain* ***≥****5%:* OR 1.13 (0.92, 1.39)  **Blue collar, retirement compared to continued working**:  *Weight loss* ***≥****5%:* OR 0.88 (0.57, 1.37)  *Weight gain* ***≥****5%:* OR 1.58 (1.13, 2.21)*  **Other/Farmer/Military, retirement compared to continued working:**  No results reported due to small cell count.  **Among overweight/obese at baseline, retirement compared to continued working:**  *Weight loss* ***≥****5%:* OR 1.15 (0.91, 1.45)  *Weight gain* ***≥****5%:* OR 1.19 (0.93, 1.52)  **Among normal weight at baseline, retirement compared to continued working:**  *Weight loss* ***≥****5%:* OR 0.81 (0.53, 1.26)  *Weight gain* ***≥****5%:* OR 1.30 (1.01, 1.69)*  * p<0.05 | **White collar, retirement compared to continued working:**  *Weight loss* ***≥****5%:* OR 1.08 (0.79, 1.47)  *Weight gain* ***≥****5%:* OR 0.85 (0.65, 1.10)  **Blue collar, retirement compared to continued working:**  *Weight loss* ***≥****5%:* OR 0.93 (0.72, 1.21)  *Weight gain* ***≥****5%:* OR 1.05 (0.83, 1.34)  **Other/Farmer/Military, retirement compared to continued working:**  *Weight loss* ***≥****5%:* OR 1.55 (0.41, 5.79)  *Weight gain* ***≥****5%:* OR 0.79 (0.23, 2.75)  **Among overweight/obese at baseline, retirement compared to continued working:**  *Weight loss* ***≥****5%:* OR 0.97 (0.79, 1.19)  *Weight gain* ***≥****5%:* OR 0.93 (0.77, 1.12)  **Among normal weight at baseline, retirement compared to continued working:**  *Weight loss* ***≥****5%:* OR 1.19 (0.76, 1.86)  *Weight gain* ***≥****5%:* OR 1.03 (0.75, 1.40) |
| Zheng 2008 [14] | **Physically demanding job, retirement compared to not retired**:  0.148 kg/m^2^ (n.s.)  **Sedentary job, retirement compared to not retired:**  0.027 kg/m^2^ (n.s.) | **Physically demanding job, retirement compared to not retired**:  0.009 kg/m^2^ *  **Sedentary job, retirement compared to not retired:**  -0.315 kg/m^2^ (p<0.01)  **Predicted 10-yr gain, with age effect**:  Physically demanding job: 0.87 kg/m^2^  Sedentary job: 0.1 kg/m^2^  * Interaction term between job type and retirement is significant at p< 0.01 |
| Chung et al. 2009 [52] | **General, retirement compared to currently working, controlled for sex:** 0.242kg/m^2^ (p<0.05)  **Physically demanding job, retirement compared to currently working, controlled for sex**: 0.478 kg/m^2^ (p<0.05)  **Sedentary job, retirement compared to currently working, controlled for sex:** 0.0432 kg/m^2^ (n.s.) | |
| Gueorguieva et al. 2011 [55] | **Professional and managerial, retirement:** Hierarchical model slope: 0.04 kg/m^2^ per year (n.s.)  **Sales, clerical and admin, retirement:** Hierarchical model slope: 0.05 kg/m^2^ per year (n.s.)  **Service, retirement:** Hierarchical model slope: 0.12 kg/m^2^ per year*  **Other blue-collar, retirement:** Hierarchical model slope: 0.13 kg/m^2^ per year#  * Slope is statistically different from the slope of the professional and managerial retirement category at p<0.05  # Slope is statistically different from the slope of the professional and managerial retirement category at p<0.01 | |
| Monsivais et al. 2015 [12] | BHPS (M & F):  **Remained Employed**: 0.60 kg/year (0.53, 0.68)  **Entered retirement:** 0.67 kg/year (0.09, 1.24)  **Lost job**: 1.56 kg/year (0.89, 2.23) | |
|  | EPIC-Norfolk (F):  **Remained Employed:**  0.42 kg/year (0.24, 0.59)  **Entered retirement:**  0.48 kg/year (0.28, 0.68)  **Lost job:**  0.69 kg/year (0.46, 0.92)*  * Weight gain among women who lost their job was statistically different from women who remained employed (p =0.007). | EPIC-Norfolk (M):  **Remained Employed:**  0.63 kg/year (0.47, 0.79)  **Entered retirement:**  0.52 kg/year (0.34, 0.70)  **Lost job:**  0.68 kg/year (0.43, 0.92)  Weight gain among men who lost their job was not statistically difference from men who remained employed. |
| Godard 2016 [56] | **Retirement compared to continuously employed:**  +0.31 kg/m^2^ (n.s.)  3.7% increase in the probability of being overweight or obese (n.s.)  2.6% increase in the probability of being obese (n.s.)  **Strenuous job, retirement compared to continuously employed**:  +0.77 kg/m^2^ (n.s.)  0.7% increase in the P(OW/OB) (n.s.)  5.1% increase in the P(OB) (n.s.)  **Sedentary job, retirement compared to continuously employed**:  +0.73 kg/m^2^ (n.s.)  8.5% increase in the P(OW/OB) (n.s.)  8.8% increase in the P(OB) (n.s.) | **Retirement compared to continuously employed:**  +0.39 kg/m^2^ (n.s.)  4.3% increase in the probability of being overweight or obese (n.s.)  11.5% increase in the probability of being obese (p<0.05)  **Strenuous job, retirement compared to continuously employed**:  +0.88 kg/m^2^ (n.s.)  6.1% increase in the P(OW/OB) (n.s.)  26% increase in the P(OB) (p< .05)*  **Sedentary job, retirement compared to continuously employed**:  +0.42 kg/m^2^ (n.s.)  5.0% increase in the P(OW/OB) (n.s.)  16.2% increase in the P(OB) (n.s.)  * Interaction term between job type and retirement is significant at p< .05 |
| Stenholm et al. 2017 [53] | **During retirement transition:**  0.15 kg/m^2^ (0.10, 0.20)  *Obesity after vs before*: RR 1.15 (1.09, 1.21)  **Sedentary, retirement transition:**  0.02 kg/m^2^ (−0.12, 0.16)  *Obesity after vs before*: RR 1.10 (0.96, 1.26)  **Diverse, retirement transition:**  0.16 kg/m^2^ (0.09, 0.22)  *Obesity after vs before*: RR 1.15 (1.07, 1.22)  **Physically heavy, retirement transition:**  0.30 kg/m^2^ (0.15, 0.46)  *Obesity after vs before*: RR 1.20 (1.07, 1.34)  **Post-retirement transition:**  0.11 kg/m^2^ (-0.01, 0.23)  *Obesity after vs before*: RR 1.00 (0.92, 1.09)  **Sedentary, post-retirement transition**  0.03 kg/m^2^ (-0.38, 0.43)  *Obesity after vs before*: RR 0.76 (0.55, 1.04)  **Diverse, post-retirement transition**  0.09 kg/m^2^ (-0.05, 0.23)  *Obesity after vs before*: RR 1.02 (0.92, 1.13)  **Physically heavy, post-retirement transition:**  0.25 kg/m^2^ (-0.04, 0.53)  *Obesity after vs before*: RR 1.08 (0.90, 1.29) | **During retirement transition:**  -0.11 kg/m^2^ (-0.22, -0.01)  *Obesity after vs before*: RR 0.93 (0.84, 1.03)  **Sedentary, retirement transition:**  −0.19 kg/m^2^ (−0.32, −0.07)  *Obesity after vs before*: RR 0.88 (0.75, 1.03)  **Diverse, retirement transition:**  −0.03 kg/m^2^ (−0.18, 0.12)  *Obesity after vs before*: RR 0.96 (0.84, 1.10)  **Physically heavy, retirement transition:**  −0.19 kg/m^2^ (−0.64, 0.26)  *Obesity after vs before*: RR 0.98 (0.71, 1.35)  **Post-retirement transition:**  0.07 kg/m^2^ (-0.14, 0.28)  *Obesity after vs before*: RR 1.05 (0.88, 1.25)  **Sedentary, post-retirement transition:**  0.08 kg/m^2^ (-0.23, 0.38)  *Obesity after vs before*: RR 1.11 (0.83, 1.48)  **Diverse, post-retirement transition:**  0.13 kg/m^2^ (-0.16, 0.43)  *Obesity after vs before*: RR 1.01 (0.77, 1.31)  **Physically heavy, post-retirement transition:**  -0.22 kg/m^2^ (-0.91, 0.46)  *Obesity after vs before*: RR 1.06 (0.73, 1.55) |
| Syse et al. 2017 [57] | **Retirement compared to those still working:**  *Weight gain of ≥ 1kg:* OR 0.78 (0.47, 1.31)  *Weight loss o*f ≥ 1kg: OR 1.75 (1.12, 2.71)  Results relevant to weight for involuntary and voluntary retirees were described as similar (data not reported).  No employment transition and sector of employment stratified results. | |
| Feng et al. 2020 [54] | **Retirement compared to non-retirees:**  BMI: 0.252 kg/m^2^ (n.s.)  Weight: -0.058 kg (n.s.) | **Retirement compared to non-retirees:**  BMI: 0.918 kg/m^2^ (p<0.05)  Weight: 2.099 kg (p<0.10) |
| Pedron et al. 2020 [51] | **Retirement compared to not retired:**  0.822 kg/m^2^ (p<0.05)  **Early retirement (at age 60) compared to not retired:**  1.514 kg/m^2^ (p<0.01)  **Delayed retirement (at age 65) compared to not retired:**  0.058 kg/m^2^ (n.s.)  **Retirement compared to employed:**  1.053 kg/m^2^ (p<0.01)  **Early retirement (at age 60) compared to employed:**  1.300 kg/m^2^ (p<0.01)  **Delayed retirement (at age 65) compared to employed:**  -0.409 kg/m^2^ (n.s.) | **Retirement compared to not retired:**  0.067 kg/m^2^ (n.s.)  **Early retirement (at age 60) compared to not retired:**  0.730 kg/m^2^ (n.s.)  **Delayed retirement (at age 65) compared to not retired:**  -0.330 kg/m^2^ (n.s.)  **Retirement compared to employed:**  0.420 kg/m^2^ (n.s.)  **Early retirement (at age 60) compared to employed:**  0.514 kg/m^2^ (n.s.)  **Delayed retirement (at age 65) compared to employed:**  0.243 kg/m^2^ (n.s.) |

*Note*. n.s. indicates a not statistically significant result. Results are based on fully-adjusted models from each study. See Table S4 for covariates used in each study.
