# Supplementary material for: A systematic review of evidence on employment transitions and weight change by gender in ageing populations
Source: PLoS One. 2022 Aug 18;17(8):e0273218. doi: 10.1371/journal.pone.0273218 (PMC9387864; doi:10.1371/journal.pone.0273218)
Supplement: S4 Table — (DOCX) [file pone.0273218.s005.docx]

**S4 Table. Covariates used for main model in included studies.**

| Study | Covariates |
| --- | --- |
| Morris et al. 1992 [16]  [ProQuest] | **Age:** continuous, years  **Social class**: 6 categories based on classes from the Office of Population Censuses and Surveys  **Town of residence** |
| Nooyens et al. 2005 [18]  [MEDLINE] | **Age:** continuous, years  **Type of job**: active or sedentary  Also, interaction term between retirement and type of job  **Smoker**: smoker or non-smoker  **Ex-smoker**: ex-smoker or non-smoker  **Started smoking**: started smoking vs non-smoker  **Bicycling duration:** hours per week  **Doing odd jobs duration:** hours per week  **Potato consumption:** times per week  **Fruit consumption:** times per week  **Breakfast consumption:** times per week  **Soft drink consumption:** glasses per day  **Fibre density:** grams per megajoule |
| Forman-Hoffman et al. 2008 [17]  [MEDLINE] | **Age:** continuous, years  **Marital status:** married or not currently married  **Race/ethnicity:** White/Caucasian, Black or African American, non-Black Hispanic, or other  **Education**: 0 to 11 years, high school graduate, some college, college graduate, or post college  **Occupational category:** white collar, blue collar, or other/farmer/military  **Smoking status:** current smoker or current non-smoker  **Alcohol consumption:** heavy drinker or not heavy drinker based on 3 or more drinks per day  **Time period**: indicator for period between study waves  **Alive status throughout all periods:** yes/no  **Baseline functional limitations:** 0, or 1 or more limitations based on eight yes/no questions  **Medical conditions:** 0, 1, or 2 or more conditions  **Depressive symptoms:** continuous, CES-D score 0 – 8  **Baseline weight category:** normal, overweight, or obese based on BMI  **Physical activity:** vigorous activities 3 times per week or more, or less than 3 times a week |
| Zheng 2008 [14]  [ProQuest] | **Age and age squared**: continuous, years  **Log household income**: continuous, in 1998 dollars  **Log household wealth**: continuous, in 1998 dollars  **Non-positive wealth:** 0/1 dummy variable  **Strenuous occupation:** 0/1 dummy variable  **White:** 0/1 dummy variable  **Hispanic:** 0/1 dummy variable  **Less than high school degree:** 0/1 dummy variable  **High school degree:** 0/1 dummy variable  **Some college without degree:** 0/1 dummy variable  **Three dummy variables for each of the following Census regions:** Northeast, Middle west, and West  **Number of instrumental activities of daily living (ADL) limitations:** continuous, range 0 – 3 limitations  **Number of ADL limitations:** continuous, 0 – 5 limitations  **Self-rated health is fair/poor**: 0/1 dummy variable  **Six dummy variables for an ever diagnosis of each of the following:**  cancer, diabetes, heart disease, hypertension, lung disease, and stroke |
| Chung et al. 2009 [52]  [MEDLINE] | **Six dummy variables for a recent health shock (past 2 years) in the following areas:**  cardiovascular, severe diabetes, cancer, lung disease, ADL limitation, and 3+ day hospitalization  **Physical activity:** yes/no to participation in vigorous physical activity or exercising 3+/week in the past 12 months  **Smoking:** currently smoker or currently non-smoker  **Age and age-squared**: continuous, years  **Female sex:** 0/1 dummy variable  **Non-Hispanic White**: 0/1 dummy variable  **College education**: 0/1 dummy variable  **High school education**: 0/1 dummy variable  **Occupation type is physically demanding**: 0/1 dummy variable  **Income/100,000:** Household income subtracted by individual’s income and divided by 100,000  **Wealth/100,00:** Household wealth divided by 100,000  **Spouse is retired:** 0/1 dummy variable  **Spouse has any ADL limitations:** 0/1 dummy variable  **Divorced**: 0/1 dummy variable  **Widowed:** 0/1 dummy variable  **New marriage:** 0/1 dummy variable whether the length of marriage is 5 years or less  **Unmarried:** 0/1 dummy variable  **Pension eligibility, social security eligibility, and spouse pension eligibility** were instrumental variables in study design**.** |
| Gueorguieva et al. 2011 [55]  [Hand search] | **Age:** continuous, years  **Gender:** woman or man  **Marital status:** married/partnered or not married  **Race/ethnicity:** White, Hispanic, Black, or other  **Years of education:** continuous, years  **Smoking status:** smoker or non-smoker  **Non-housing-related wealth:** five levels of wealth (lowest: ≤ $7,500; highest: over $150,000)  **Health insurance:** employer-sponsored, no employer-sponsored, government-sponsored, no government-sponsored, other private, or no other private  **Hours worked per week before retirement:** continuous, hours  **Dropout status:** completer, deceased during study, or dropped out during study |
| Monsivais et al. 2015 [12]  [MEDLINE] | **Age**: continuous, years  **Sex**: woman or man  **Smoking status**: current smoker, former smoker, or never smoker  **Baseline weight**: baseline weight in kilograms  **Educational attainment**: no qualifications, O-level, A-level, or degree  Sex-stratified models additionally included:  **Change in smoking status**: remained non-smoker, quit smoking, started smoking, or remained a smoker  **Change in dietary energy intake**: continuous, megajoules per day  **Change in physical activity**: decrease, increase, or no change |
| Godard 2016 [56]  [Hand search] | **Age and age squared**: continuous, years  **Marital status**: lives with a spouse/partner or does not live with a spouse/partner  **Gender:** woman or man  **Educational level:** primary education, lower secondary, upper secondary, or post secondary  **Occupation:** blue collar, white collar, technician, manager, or professional  **Country**: Austria, Belgium, France, Germany, Italy, Spain, Sweden, or Switzerland  **Self-reported health**: five-point scale from excellent to poor  **Euro-D** **depression index**: continuous, 0 – 12 with higher scores indicating greater feelings of depression |
| Stenholm et al. 2017 [53]  [MEDLINE] | **Retirement age**  **Socioeconomic position:** high, intermediate, or low based on International Standard Classification of Occupations  **Physical activity:** metabolic equivalent of energy expenditure on activity duration and intensity level  **Alcohol use:** grams of alcohol per week  **Smoking:** never smoker, former smoker, or current smoker  **Marital status:** not defined in article  **BMI:** continuous, kg/m^2^  **Number of chronic diseases:** 0, 1, or 2 or more chronic diseases  **Job strain prior to retirement**: 0/1 dummy variable |
| Syse et al. 2017 [57]  [PsycINFO] | **Age:** 57-59 years, 60-62 years, or 63-66 years  **Marital status**: single or married/cohabitating  **Work related stress**: stressful work or un-stressful work  **Work hours:** <37 hrs, 37-40 hrs, or >40hrs  **Sector of employment**: public sector or private sector  **Self-reported health:** excellent/very good/good or fair/poor  **Physical health score:** continuous, based on PCS-12  **Smoking status**: former/never smoker or current smoker  **Medication use**: less than weekly or weekly |
| Feng et al. 2020 [54]  [MEDLINE] | **Rural:** 0/1 dummy variable  **Marital status:** single, widowed, or married with spouse present  **Age and age squared/100:** continuous, years  **Household size:** continuous, count |
| Pedron et al. 2020 [51]  [MEDLINE] | **Sex**: male or female  **Education**: low education or high education  **Living alone**: 0/1 dummy variable  **Age and age-squared** were the assignment variables for regression discontinuity design to create control group |
